# Cryo-EM structure reveals a symmetry reduction of the plant outward-rectifier potassium channel SKOR

**DOI:** 10.1038/s41421-023-00572-w

**Published:** 2023-06-30

**Authors:** Siyu Li, Yuanxia Wang, Chenyang Wang, Yong Zhang, Demeng Sun, Peng Zhou, Changlin Tian, Sanling Liu

**Affiliations:** 1 Department of Endocrinology, Institute of Endocrine and Metabolic Diseases, The First Affiliated Hospital of USTC, Division of Life Sciences and Medicine, Joint Center for Biological Analytical Chemistry, Anhui Engineering Laboratory of Peptide Drug, Anhui Laboratory of Advanced Photonic Science and Technology, University of Science and Technology of China, Hefei, Anhui China; 2 School of Life Science, Hefei Normal University, Hefei, Anhui China; 3 The Anhui Provincial Key Laboratory of High Magnetic Resonance Image, High Magnetic Field Laboratory, Chinese Academy of Sciences, Hefei, Anhui China

**Keywords:** Cryoelectron microscopy, Plant molecular biology

Dear Editor,

In plant, the family of voltage-gated potassium channel is crucial for K^+^ uptake, release, and distribution, playing important roles in plant growth and development^1,2^. Among these channels, the Stelar K^+^ outward rectifier channel (SKOR) functions as a K^+^ efflux channel for long-distance distribution of K^+^ from roots to the upper parts of the plant^3,4^. There are only two structures of voltage-gated K^+^ channels, KAT1 and AKT1 from *Arabidopsis*, reported to date^5–7^. Notably, KAT1/AKT1 and SKOR represent two different types of K^+^ channels that share an extensively similar structural background but display profoundly different biophysical characteristics, belonging to K_in_ and K_out_ channels, respectively^2^. Interestingly, SKOR contains an intracellular ankyrin repeat (ANK) domain, which is absent in all the depolarization-activated K^+^ channels with known structure. Here, we reported the cryo-EM structure of SKOR, and investigated the role of the ANK domain in channel structure and function modulation.

After extensive 3D classification and refinement, two final maps of SKOR (named SKOR_wt1_ and SKOR_wt2_) with global resolutions of 3.1 Å and 3.5 Å were obtained (Supplementary Figs. S1–S4 and Table S1). The overall structures of these two models show a similar architecture: a tetrameric assembly around a central pore (Fig. 1a, b). Each protomer can be arbitrarily divided into four layers along the central axis: a transmembrane domain (TMD) comprising six transmembrane α-helices designated as S1–S6, a gating ring formed by the C-linker, a cytoplasmic domain homologous with the cyclic nucleotide-binding domain (CNBD), and an ANK domain (Fig. 1c). Structural comparison shows that the tetrameric TMDs of SKOR_wt1_ and SKOR_wt2_ are almost identical (Supplementary Fig. S5a). The TMD consists of a voltage sensor domain (VSD, helices S1–S4) and a pore-forming domain (PD, helices S5 and S6). The VSD is connected to the PD through a short linker, so that the PD and VSD of the same subunit coalesce together as a bundle. Therefore, SKOR is topologically a non-domain-swapped ion channel similar to KAT1 and AKT1 channels (Fig. 1d).

**Fig. 1:**
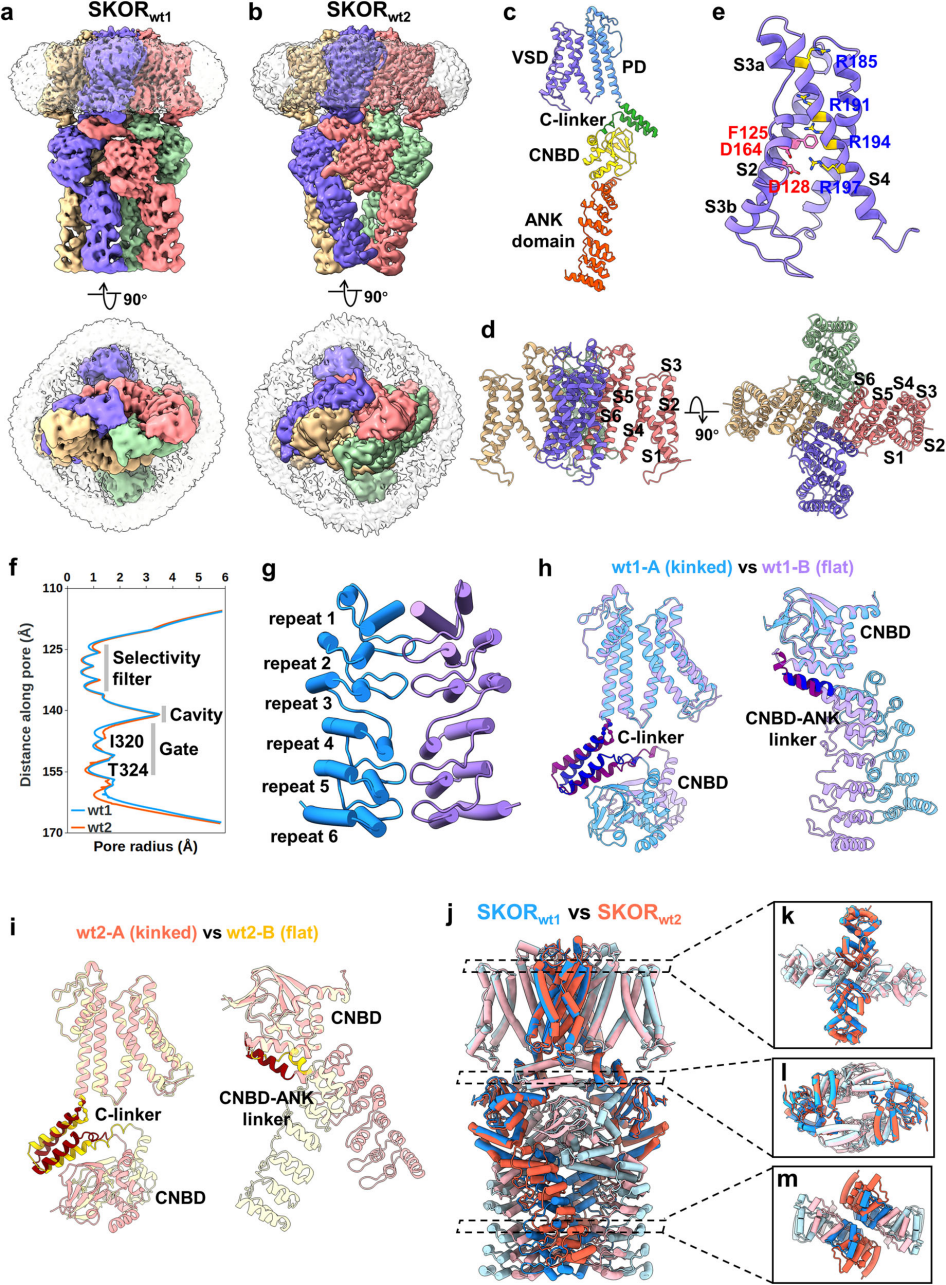
Cryo-EM structures of SKOR from *Arabidopsis*. **a**, **b** Two different cryo-EM maps of SKOR were segmented and colored according to four subunits. The detergent micelles were shown as transparent surface. **c** Cartoon representation of a single subunit of SKOR. **d** Cartoon representation of the tetrameric TMD of SKOR reveals a non-domain-swapped architecture. **e** The deactive voltage-sensing domain with positively charged residues (Arg185, Arg191, Arg194, Arg197) and charge transfer center residues (Phe125, Asp128 and Asp164) shown. **f** Channel pore radius along the ion conduction pathway of SKOR calculated using HOLE. **g** Cartoon representation of a dimeric ANK domain. **h**, **i** The C-linkers and CNBD–ANK linkers from two adjacent subunits adopt different conformations in both SKOR_wt1_ and SKOR_wt2_. **j** Structural comparison of SKOR_wt1_ and SKOR_wt2_ reveals a rotation in the ANK domains. **k**–**m** Top views of the tetrameric TMDs, C-linkers, CNBDs and ANK domains of SKOR_wt1_ and SKOR_wt2_. Symmetry reduction from C4 to C2 is shown.

In VSD, four positively charged residues are located on the S4 helix, and F125 at S2 helix forms the hydrophobic constriction site (HCS)^8,9^. The residues R185, R191 and R194 are located above the HCS, whereas R197 is below the HCS closely interacting with an intracellular negative cluster formed by D128 and D164 (Fig. 1e). This indicates that the S4 helix is in a resting, “up” conformation. In PD, the P-loop segment, consisting of Thr–Val–Gly–Tyr–Gly residues, is consistent with the universal K^+^-selective TxGYG motif (Supplementary Fig. S5b). Under the filter, S6 helices pack to form the channel pore and the gate. The gate of SKOR constricts the pore to a radius of ~1 Å at positions Ile320 and Thr324, precluding the diffusion of ions through the central pore (Fig. 1f; Supplementary Fig. S6a). The resting conformation of S4 helix, together with the closed gate formed by S6 helices, indicates that the SKOR structure is in a closed state.

Following the TMD, four copies of the C-linker form an α-helical disk at the intracellular membrane–cytoplasm interface. CNBDs following the C-linker are docked onto the cytoplasmic face of the C-linker disk. Structural comparison of the C-linkers and CNBDs from SKOR_wt1_ and SKOR_wt2_ reveals subtle differences (Supplementary Fig. S5a). Compared to KAT1, SKOR_wt1_ exhibits a significant rotation in the C-linker and CNBD. Moreover, the distinct extent of rotation in the adjacent subunits leads to breaking of C4 symmetry in SKOR (Supplementary Fig. S6b). Following the CNBD, six ANKs fold as a single ANK domain, which adopts a slightly curved solenoid conformation. Among these repeats, the repeats 1, 2, 4 and 5 are canonical and display the characteristic α-helix/α-helix/β-hairpin structure, whereas the repeats 3 and 6 lack the β-hairpin (Fig. 1g). ANKs are present in many plant or mammalian proteins, including the transient receptor potential ankyrin 1 (TRPA1) ion channel. The cryo-EM structure of TRPA1 shows five well-defined ANKs in its N-terminus^10^. Superposition of the structures of the ANKs from SKOR_wt1_ and TRPA1 reveals that repeats 1 and 2 in SKOR are assembled similarly to those in TRPA1, whereas repeats 4, 5 and 6 display a clockwise rotation relative to TRPA1 due to the lack of β-hairpin in repeat 3.

Interestingly, the overall architectures of both SKOR_wt1_ and SKOR_wt2_ do not adopt canonical C4 symmetry but exhibit a dramatic reduction of symmetry from C4 to C2. The TMD adopts a C4 symmetry configuration, while the domains following the TMD, including the C-linker, CNBD and ANK adopt C2 symmetry (Supplementary Fig. S5c). Structural comparison of two adjacent subunits of both SKOR_wt1_ and SKOR_wt2_ reveals different conformations (Supplementary Fig. S5d). Further alignment shows that the individual domains in each two neighboring subunits have almost identical structures, while the C-linker and CNBD–ANK linker display obviously different conformations (Fig. 1h, i). The C-linkers from two neighboring subunits (A and B) adopt distinct configurations: a “flat” conformation similar to that observed in the structure of HCN1, and a “kinked” conformation similar to that in KAT1. The subunit A containing the kinked C-linker has a kinked CNBD–ANK linker. In contrast, the adjacent subunit B shows a flat CNBD–ANK linker. Taking together, the conformational heterogeneities in C-linker and CNBD–ANK linker in the adjacent subunits might result in the symmetry reduction.

The packing of ANKs in SKOR_wt2_ is different from that in SKOR_wt1_. The superimposition of these two structures shows only subtle differences in TMD and CNBD (Fig. 1j–l) but a significant rotation in the ANK domains (Fig. 1m). This observation indicates that the configuration of ANKs in SKOR is dynamic. The twisting of ANKs in SKOR_wt2_ leads the CNBD and ANK domain to move closer to each other, resulting in a more compact architecture compared to SKOR_wt1_.

This symmetry reduction is uncommon and has not been observed in other voltage-gated K^+^ channels lacking ANK. Recently reported structure of AKT1, which also has an ANK domain, exhibits a similar symmetry reduction^5^, suggesting that ANKs may play a critical role in this process. In the structure of SKOR_wt1_, four ANK domains are divided into two pairs; the two ANK domains in each pair form close contacts with each other, and a gap is present between the two pairs, resulting in a C2 symmetry. As a result of this asymmetry, the inter-CNBD interactions around the channel ring differ considerably. Previous studies have demonstrated that CNBDs in plant Shaker-like channels probably lose the ligand-binding activity^5,6^. Our electrophysiological data showed no obvious effect of additional cNMP on SKOR current (Supplementary Fig. S7a). The arginine residue (R549) important for cAMP binding in CNBD of HCN1 is substituted by glutamine (Q482) in SKOR (Supplementary Fig. S7b). Structural comparison of CNBDs in the HCN1/cAMP complex and SKOR indicates potential clashes between cAMP and the side chain of Q482 in SKOR (Supplementary Fig. S7c), which is in consistent with the functional results. Notably, as a depolarization-activated channel, SKOR was expected to adopt an open conformation in vitro (0 mV) like Kv1.3^11^ and hERG channels^12^, but both SKOR_wt1_ and SKOR_wt2_ were found to be in a closed state. The ANK-induced symmetry reduction may be responsible for this closed state.

Some studies have suggested that ANK phosphorylation may be involved in regulating the activity of K^+^ channels. For instance, a protein complex consisting of calcineurin B-like protein (CBL) and CBL-interacting protein kinase (CIPK) was reported to phosphorylate the ANKs of GORK, an outward potassium channel in *Arabidopsis*, to activate K^+^ transport^13,14^. However, when CIPK alone or in pairs with CBL were co-expressed with SKOR, no direct influence on SKOR current was found (Supplementary Fig. S8a, b). Mutations of serine or threonine residues in the ANK domain did not significantly affect channel activity, suggesting that these sites may not contribute to ANK phosphorylation in SKOR (Supplementary Fig. S8c, d). Further studies are needed to investigate the regulation of SKOR activity via ANK.

Besides the wild-type SKOR, we also determined the structure of an SKOR mutant L271P-D312N (SKOR_mut_), which was reported to convert outwardly rectifying SKOR into an inward rectifier^15^, at a resolution of 3.1 Å (Supplementary Figs. S9–S11). Structural comparison of SKOR_wt1_ and SKOR_mut_ showed that the pore domains of these two structures are almost identical (Supplementary Fig. S12), indicating that SKOR_mut_ is also in a closed state. Notably, our electrophysiological data demonstrated that SKOR_mut_ is a non-functional channel (Supplementary Fig. S9a), rather than a converted inward rectifier, which was consistent with the closed structure in vitro.

In summary, the cryo-EM structures of SKOR provide valuable insights into the architecture of depolarization-activated K^+^ channels with an ANK domain. The tetrameric assembly of SKOR around a central pore, the TMD, C-linker, CNBD, and ANK domain, as well as their interactions, are well characterized. Unlike the canonical potassium channel, SKOR does not adopt a C4 but a C2 symmetry, which occurs in the C-linker, CNBD and ANK domain. The reduction in symmetry suggests a unique regulatory mechanism for SKOR activation. Together, this study paves the way for further investigations of the functional properties and physiological roles of SKOR and other depolarization-activated K^+^ channels with an ANK domain.

## Supplementary information

Supplementary Information

## Data Availability

Cryo-EM density maps and models for SKOR_wt1_, SKOR_wt2_ and SKOR_mut_ have been deposited in the Electron Microscopy Data Bank (EMDB) and the Protein Data Bank (PDB). The EMDB accession codes are EMD-36195 (SKOR_wt1_), EMD-36196 (SKOR_wt2_), EMD-36185 (SKOR_mut_), EMD-36197 (SKOR_wt1-ICD_), EMD-36198 (SKOR_wt2-ICD_) and EMD-36199 (SKOR_mut-ICD_); and the PDB accession codes are 8JET (SKOR_wt1_), 8JEU (SKOR_wt2_) and 8JEC (SKOR_mut_).
