# Supplementary material for: Cryo-EM structure reveals a symmetry reduction of the plant outward-rectifier potassium channel SKOR
Source: Cell Discov. 2023 Jun 30;9:67. doi: 10.1038/s41421-023-00572-w (PMC10313817; doi:10.1038/s41421-023-00572-w)
Supplement: Supplementary file 1 — Supplementary Information [file 41421_2023_572_MOESM1_ESM.pdf]

Cryo-EM structure reveals a symmetry reduction of the plant outward-rectifier potassium channel SKOR

Siyu Li[1], Yuanxia Wang[1], Chenyang Wang[1], Yong Zhang[1], Demeng Sun[1], Peng Zhou[2], Changlin Tian[1,3] and Sanling Liu[1]

[1]Department of Endocrinology, Institute of Endocrine and Metabolic Diseases, The First Affiliated Hospital of USTC, Division of Life Sciences and Medicine, Joint Center for Biological Analytical Chemistry, Anhui Engineering Laboratory of Peptide Drug, Anhui Laboratory of Advanced Photonic Science and Technology, University of Science and Technology of China, Hefei 230026, China

[2]School of Life Science, Hefei Normal University, Hefei 230601, China

[3]The Anhui Provincial Key Laboratory of High Magnetic Resonance Image, High Magnetic Field Laboratory, Chinese Academy of Sciences, Hefei 230031, China

These authors contributed equally: Siyu Li, Yuanxia Wang, Chenyang Wang

Correspondence: Peng Zhou (pp7196@126.com) or Changlin Tian (cltian@ustc.edu.cn) or Sanling Liu (sanling@ustc.edu.cn)

Supplementary Information

Materials and Methods

Protein expression and purification

The optimized coding DNAs for SKOR (Uniprot: Q9M8S6) and KAB1 (Uniprot: O23016) from *Arabidopsis thaliana* were synthesized by Sangon Biotech (Shanghai). The SKOR and KAB1, a component of some plant K^+ channels, were cloned into a pFastBac-Dual vector with a FLAG tag (DYKDDDDK) at the amino terminus of SKOR. Baculovirus-infected *Sf9* cells (Thermo Fisher) were used for overexpression and were grown at 27 °C in SIM SF Expression Medium (Sino Biological Inc.). Transfected cells were cultured for 60 h before harvesting.

Cell pellet from 1 L of culture was resuspended in extraction buffer (10 mM lauryl maltose neopentyl glycol (LMNG), 2 mM cholesteryl hemisuccinate (CHS), 300 mM KCl, 20 mM Tris pH 8.0 and protease inhibitor cocktail (Roche)) at 4°C for 1.5 hours. Solubilized membranes were clarified by centrifugation at 20,000 × g for 30 min at 4°C. The supernatant was applied to anti-Flag M2 affinity gel (Sigma) by gravity at 4 °C. The resin was rinsed four times with the wash buffer (0.04% GDN, 300 mM KCl and 20 mM Tris pH 8.0). The target proteins were eluted with wash buffer supplemented with 200 μg/ml FLAG peptide. The eluent was concentrated by Amicon Ultra centrifugal filter (MWCO 100 kDa), and then injected to a Superose 6 increase column (GE Healthcare) equilibrated with SEC buffer (0.02% GDN, 150 mM KCl, 20 mM Tris pH 8.0 and 2 mM DTT). Peak fractions were pooled and concentrated to 5 mg/ml.

Single-particle cryo-EM data acquisition

Purified protein (3 μl) at a concentration of 5 mg/ml was added to the freshly plasma-cleaned holey carbon grids (Quantifoil, R1.2/1.3, 300 mesh, Au). The grids were blotted for 6.5 s at 100% humidity and 4 °C with a Vitrobot Mark IV (ThermoFisher Scientific) and plunge-frozen into liquid ethane cooled by liquid nitrogen. The blotted grids were stored in liquid nitrogen until imaged.

Grids were transferred to a Titan Krios electron microscope (FEI) operated at 300 kV equipped with a Gatan K2 Summit direct detection camera. Images were collected using the automated image acquisition software SerialEM[1] in counting mode with 29,000 × magnification yielding a pixel size of 1.01 Å. The total dose of 56 e-/Å2 was fractionated to 32 frames. Nominal defocus values ranged from -1.0 to -2.0 μm. Datasets of SKOR and the D312N-L271P mutant included 4,293 and 1,057 movies, respectively.

Image processing

Dose-fractionated image stacks were subjected to beam-induced motion correction and dose-weighting using UCSF MotionCor2[2]. Contrast transfer function parameters were estimated with Gctf[3]. For particle picking, around 2,000 particles were picked manually to generate references for auto-picking in Relion-3[4]. The atuo-picked particles were extracted by four-times downscaling resulting in the pixel size of 4.04 Å and then subjected to reference-free 2D classification. For the dataset of SKOR, 435,204 particles from well-defined 2D averages were selected for 3D classification with a pixel

size of 2.02 Å. A 3D initial model de novo from the 2D average particles was generated using stochastic gradient descent (SGD) algorithm in Relion. The 50 Å low-pass filtered initial model was used as a reference for 3D classification into four classes. Two selected classes with continuous density for all transmembrane helices were combined and used to perform further 3D classification by heterogeneous refinement in CryoSPARC4[5]. Particles in good classes were subjected in non-uniform refinement with C2 symmetry imposed and produced the final 3D reconstructions. Two subsets of 129,992 and 62,707 particles produced final maps with global resolutions of 3.1 Å and 3.5 Å, respectively. Further local refinement with masks only focusing on the intracellular domain (ICD) produced two maps at resolution of 3.9 Å and 4 Å, respectively. For the dataset of the mutant, 197,544 particles from well-defined 2D averages were selected and combined for 3D classification. An ab initio 3D reconstruction from the 2D average particles was generated in cryoSPARC. The initial model was then used as a reference for 3D classification by heterogeneous refinement. A good class with continuous density for all transmembrane helices was subjected in non-uniform refinement with C2 symmetry imposed and produced the final 3D reconstruction with a resolution of 3.1 Å. Further local refinement with mask only focusing on the ICD produced a map at resolution of 3.6 Å. The global resolutions were estimated by applying a soft mask excluding the detergent micelle based on the gold-standard Fourier shell correlation (FSC) using the 0.143 criterion. Local resolution values were estimated in CryoSPARC4.

Model building, refinement and validation

The coordinate of KAT1 (PDB code 7ACL)[6] was fitted into the 3D EM maps of SKOR using UCSF Chimera[7]. The sequences were mutated with corresponding residues in SKOR in Coot[8]. For the ankyrin-repeats domain, the local-refined map of $SKOR_{mut}$ was used for model building with an initial model predicted by AlphaFold[9]. Every residue was manually examined. The chemical properties of amino acids were considered during model building. The model was subjected to iterative manual rebuilding in Coot and real-space refinement in PHENIX[10]. The final model was validated using the module "comprehensive validation (cryo-EM)" in PHENIX[11]. The N-terminal residues before S73 and C-terminal residues after S740 were not built due to the lack of corresponding densities. subjected to refinement and validation in PHENIX. All the figures were prepared using UCSF ChimeraX[12] and Chimera.

Electrophysiology experiments

DNA encoding SKOR was cloned into a pcDNA3.1/Zeo(+) vector. All site-directed mutations were generated with overlap PCR and inserted into pcDNA3.1/Zeo(+). All plasmids were sequenced before further study. Chinese hamster ovary (CHO) cells were cultured in DMEM/F12 medium (Gibco) supplemented with 10% fetal bovine serum (FBS) and 1% Penicillin-Streptomycin-Glutamine (GIBCO) at 37 °C in a 5% CO_2 incubator. For each transfection of a 24-well-plate well, 0.8 μg of plasmid encoding EGFP and 1 μg of plasmid encoding SKOR or the mutants (or 2 μg of plasmid encoding EGFP and SKOR) were mixed with lipofectamine 3000 transfection reagent (Invitrogen) and added to the cells. After incubation for 5 hours, the cells were transferred to poly-L-lysine (Sigma)-coated slides to culture for another

24-48 hours in fresh medium. They were then used for the electrophysiological recordings.

For whole-cell patch clamp recordings, the bath solution contained 150 mM NaCl, 4 mM KCl, 2 mM $CaCl_2$, 1 mM $MgCl_2$, and 10 mM HEPES (pH 7.4, ~308 mOsm). The electrodes were pulled from thick-walled borosilicate glass capillaries with filaments (1.5 mm diameter, Sutter Instruments) on a four-stage puller (P-1000, Sutter Instruments) and had resistances of 3-5 MΩ when filled with intracellular solution containing 140 mM KCl, 10 mM NaCl, 5 mM EGTA, 10 mM HEPES (pH 7.4, ~297 mOsm). All chemicals were obtained from Sigma. Experiments were performed at room temperature with an EPC-10 amplifier (HEKA Electronic) using the data acquisition software PatchMaster. Families of SKOR or the mutants were elicited by voltage steps from -80 mV to +100 mV in 20 mV increments for 2 s, from a holding potential of -80 mV. Tail currents were recorded at -100 mV. The pulses were applied every 20 s. Tail current values were normalized to compare current–voltage relationships between SKOR and the mutants.

References

1 Mastronarde, D. N. SerialEM: A Program for Automated Tilt Series Acquisition on Tecnai Microscopes Using Prediction of Specimen Position. *Microsc Microanal* **9**, 1182-1183 (2003).

2 Zheng, S. Q. *et al.* MotionCor2: anisotropic correction of beam-induced motion for improved cryo-electron microscopy. *Nat Methods* **14**, 331-332 (2017).

3 Zhang, K. Gctf: Real-time CTF determination and correction. *J Struct Biol* **193**, 1-12 (2016).

4 Zivanov, J. *et al.* New tools for automated high-resolution cryo-EM structure determination in RELION-3. *Elife* **7**, e42166 (2018).

5 Punjani, A., Rubinstein, J. L., Fleet, D. J. & Brubaker, M. A. cryoSPARC: algorithms for rapid unsupervised cryo-EM structure determination. *Nat Methods* **14**, 290-296 (2017).

6 Li, S. *et al.* Cryo-EM structure of the hyperpolarization-activated inwardly rectifying potassium channel KAT1 from *Arabidopsis*. *Cell Res* **30**, 1049-1052 (2020).

7 Pettersen, E. F. *et al.* UCSF Chimera--a visualization system for exploratory research and analysis. *J Comput Chem* **25**, 1605-1612 (2004).

8 Emsley, P., Lohkamp, B., Scott, W. G. & Cowtan, K. Features and development of Coot. *Acta Crystallogr D Biol Crystallogr* **66**, 486-501 (2010).

9 Jumper, J. *et al.* Highly accurate protein structure prediction with AlphaFold. *Nature* **596**, 583-589 (2021).

10 Adams, P. D. *et al.* PHENIX: a comprehensive Python-based system for macromolecular structure solution. *Acta Crystallogr D Biol Crystallogr* **66**, 213-221 (2010).

11 Afonine, P. V. *et al.* New tools for the analysis and validation of cryo-EM maps and atomic models. *Acta Crystallogr D Biol Crystallogr* **74**, 814-840 (2018).

12 Goddard, T. D. *et al.* UCSF ChimeraX: Meeting modern challenges in visualization and analysis. *Protein Sci* **27**, 14-25 (2018).

Supplementary Figures

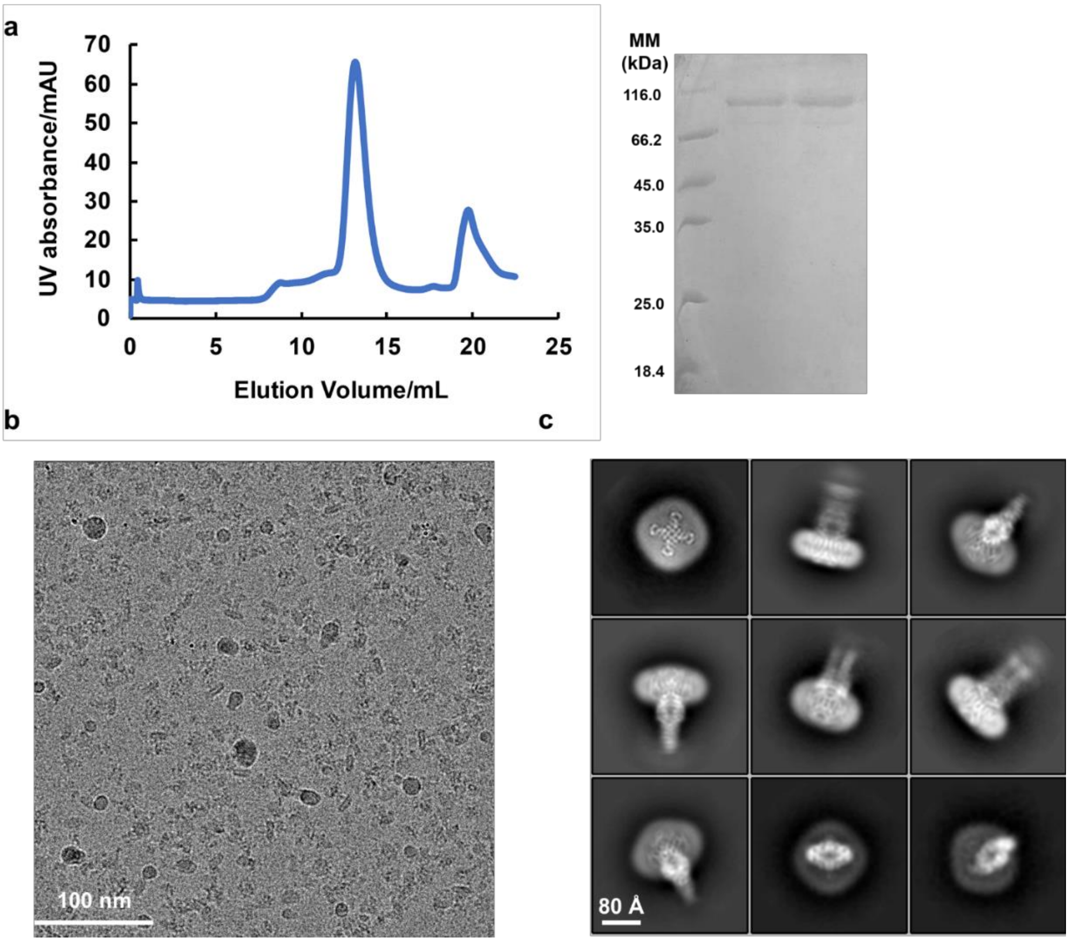

Supplementary Fig. S1. Purification and cryo-EM sample preparation of wild-type SKOR. **a**. Size-exclusion chromatography (left) and SDS-PAGE analysis (right) for purification. **b** and **c**. A representative cryo-EM micrograph (**b**) and representative 2D class averages (**c**) of $SKOR_{wt}$.

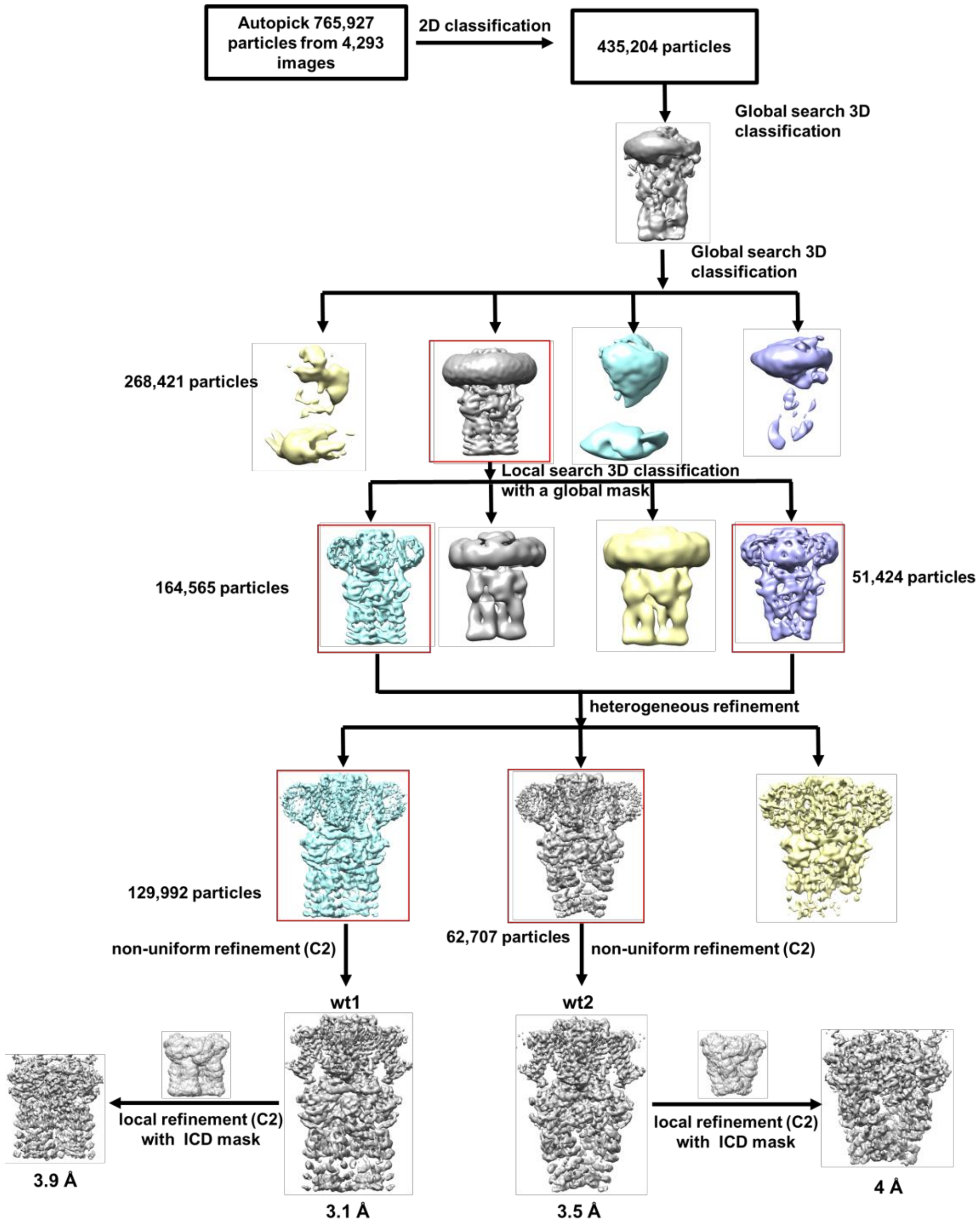

Supplementary Fig. S2. Cryo-EM data processing flow chart of wild-type SKOR.

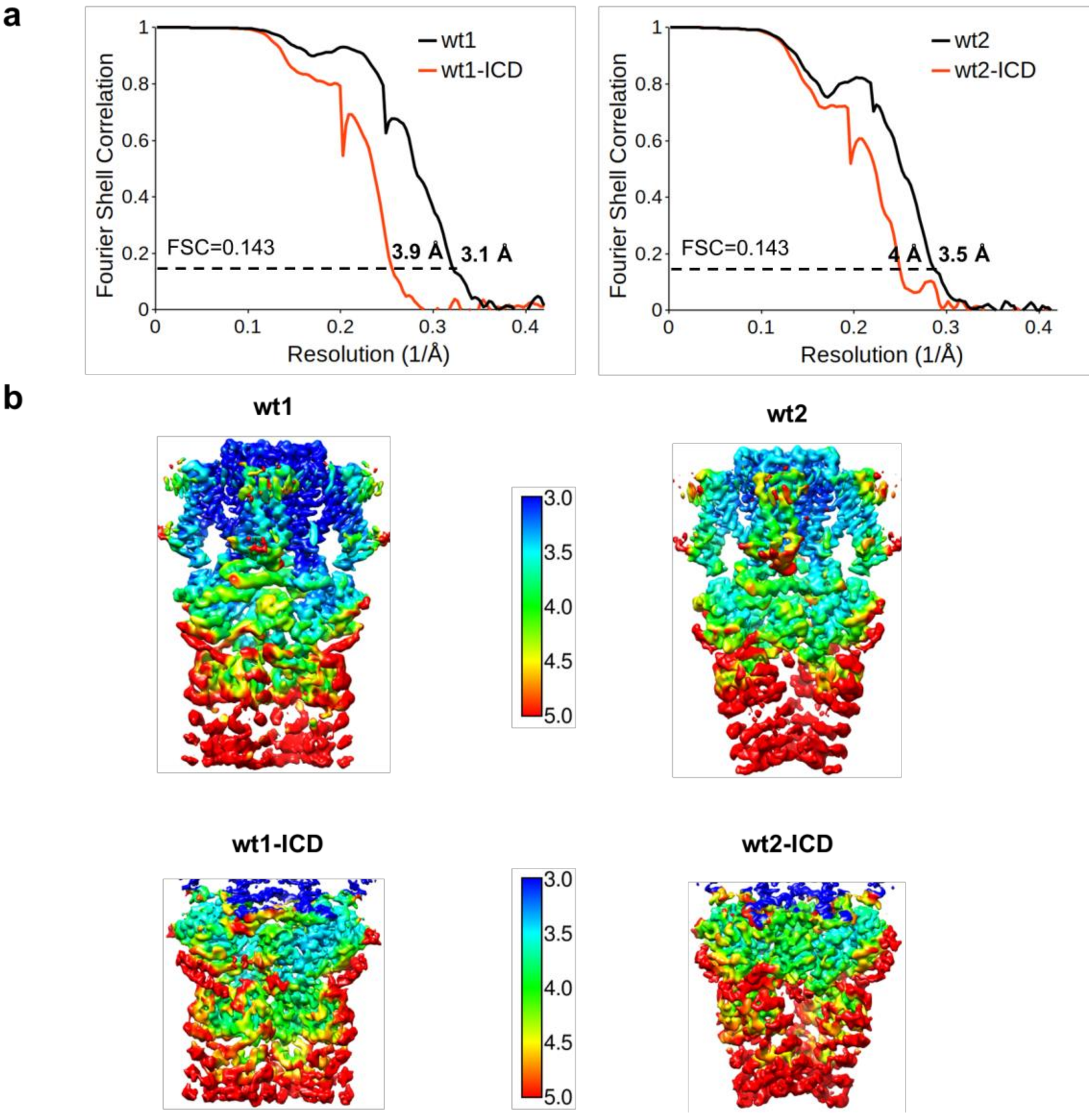

Supplementary Fig. S3. FSC curves and local resolution estimation for $SKOR_{wt1}$ and $SKOR_{wt2}$. a. Gold-standard Fourier shell correlation (FSC) curves for the 3D reconstructions of $SKOR_{wt1}$ and $SKOR_{wt2}$. **b**. EM density colored according to local resolution estimate.

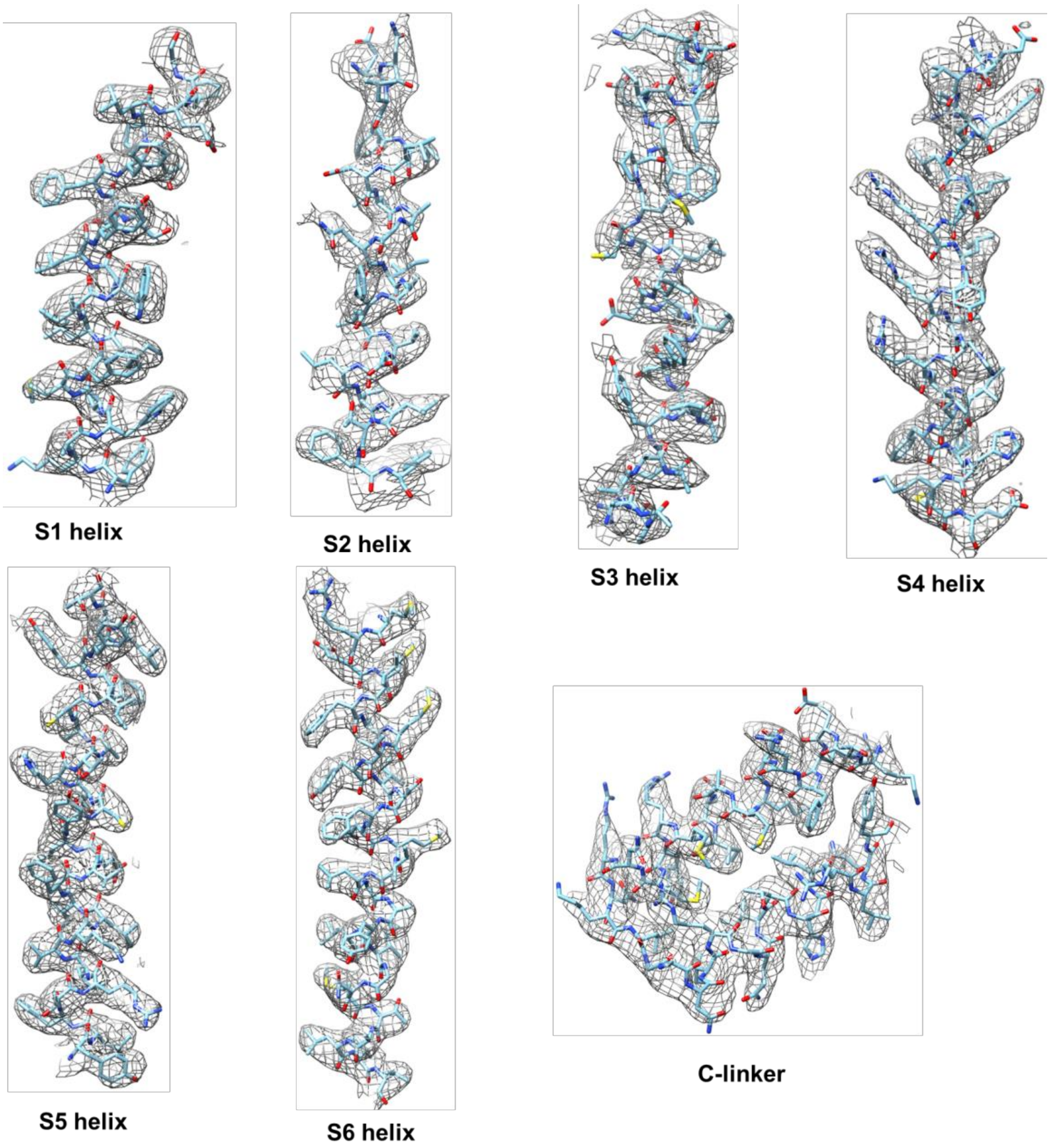

Supplementary Fig. S4. Agreement between the cryo-EM map and the model of SKOR$_{wt1}$. Representative cryo-EM densities and fitted atomic models are shown.

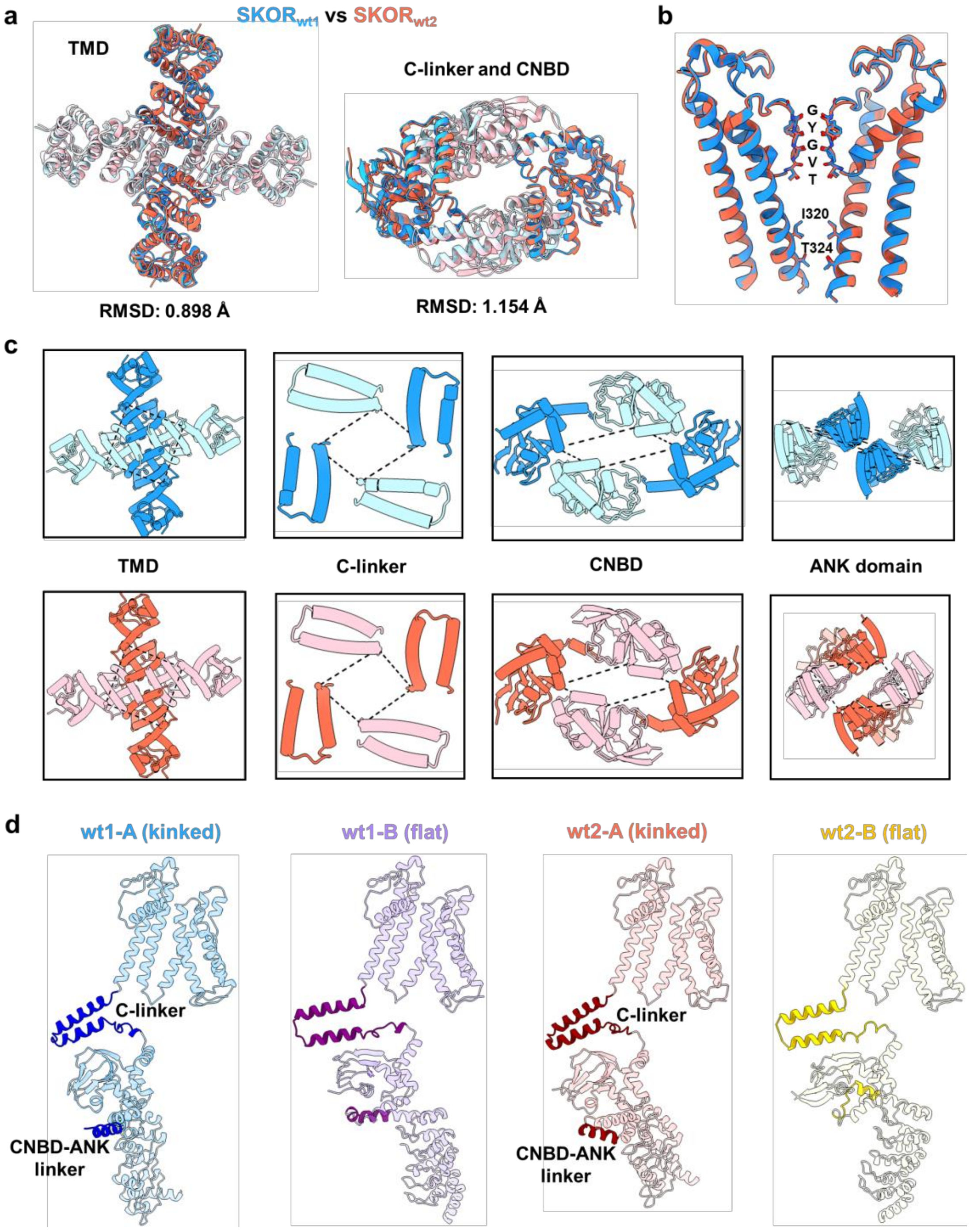

Supplementary Fig. S5. Structural comparison of SKOR$_{wt1}$ and SKOR$_{wt2}$. a. Overlay of TMD (left) and C-linker and CNBD domain (right) in SKOR$_{wt1}$ (blue) and SKOR$_{wt2}$ (red), respectively. RMSD values between corresponding Cα atom pairs are labeled. b. Structures of the pore of SKOR (wt1: blue, wt2: red). Pore-lining residues on the P-loop and S6 are shown, including the selectivity filter near the extracellular side, and the gate at the intracellular side. c. Bottom views of the tetrameric TMD, C-linker, CNBD and ANK domains of SKOR$_{wt1}$ (top) and SKOR$_{wt2}$ (bottom) show a

symmetry reduction from C4 to C2. d. Comparison of two adjacent subunits in $SKOR_{wt1}$ and $SKOR_{wt2}$. The C-linker and CNBD-ANK linker display two different conformations, which are “flat” or “kinked”.

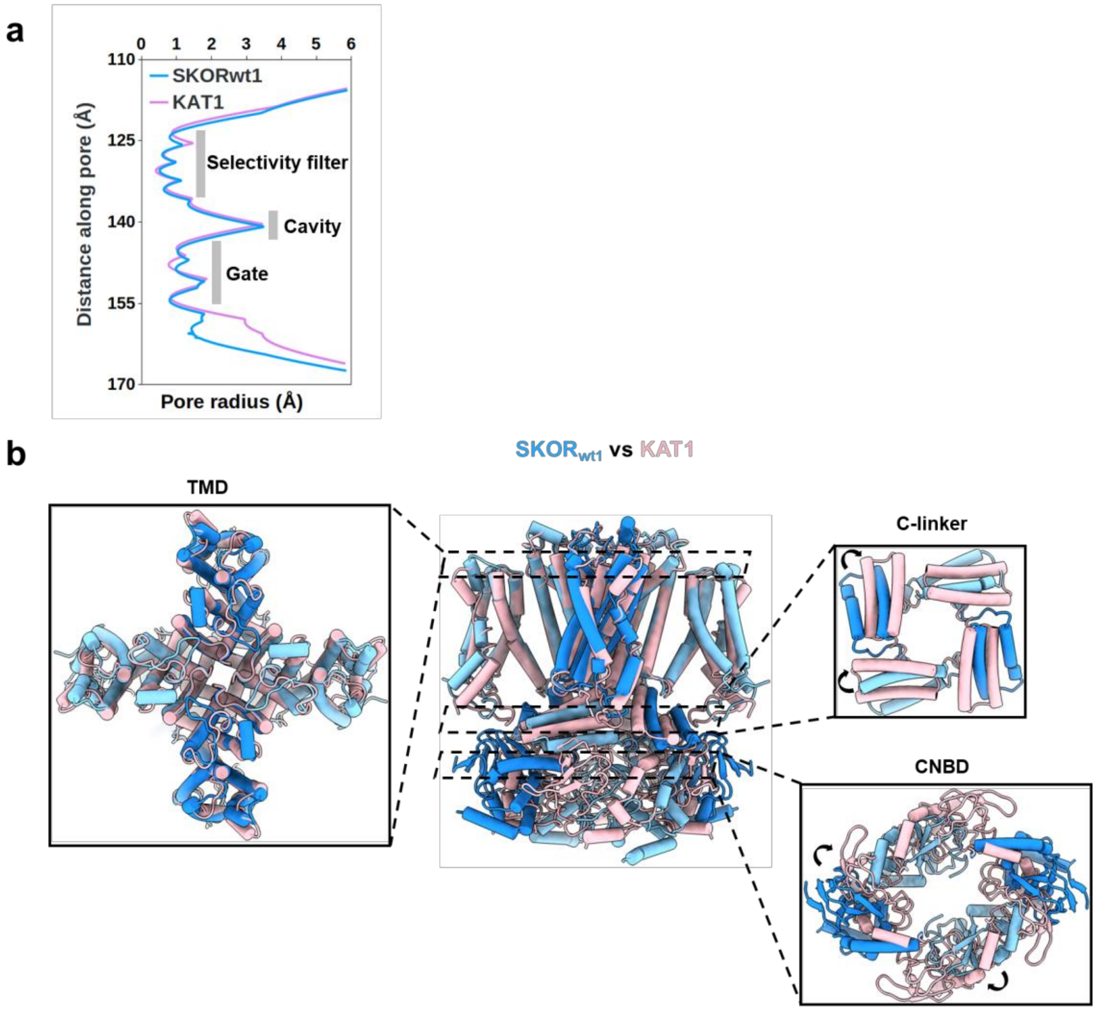

Supplementary Fig. S6. Structural comparison of $\mathbf{SKOR_{wt1}}$ and KAT1. a. Channel pore radius along the ion conduction pathway of $SKOR_{wt1}$ (blue) and KAT1 (pink) calculated using the HOLE program. b. Structural comparison of $SKOR_{wt1}$ (blue) and KAT1 (pink) reveals a rotation in the intracellular domains.

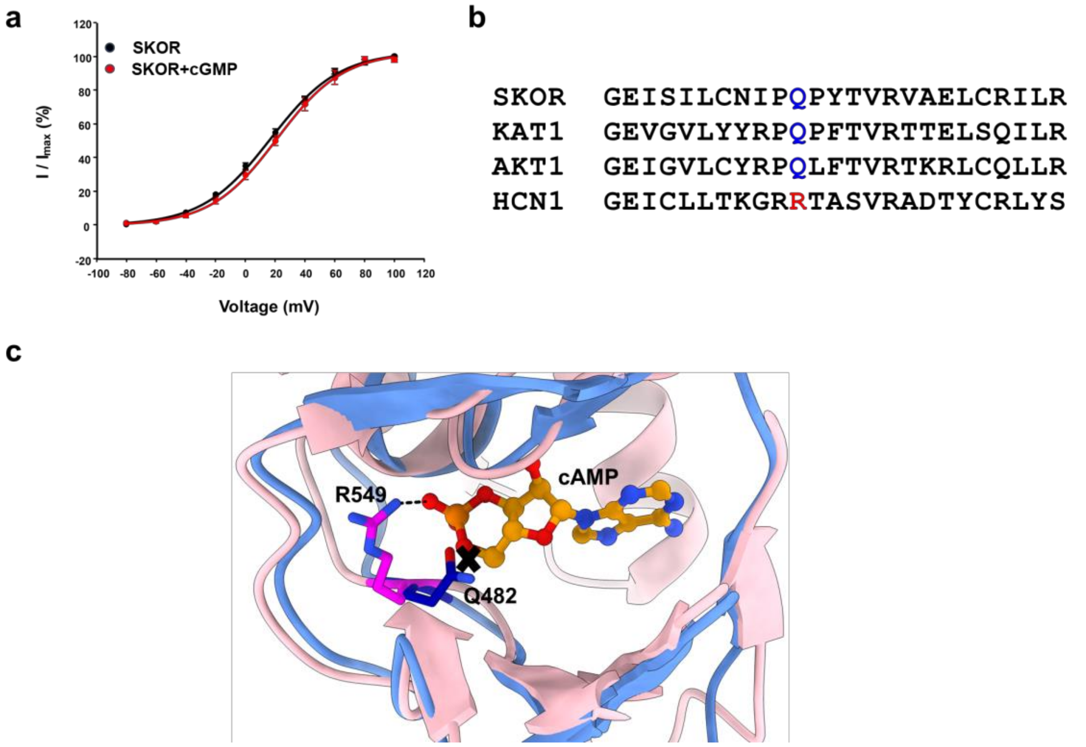

Fig S7. cNMP binding domain of SKOR channel. a. Voltage dependence of the activation of SKOR channel (black) and SKOR/cGMP (red). (means ±SE, n > 5 for each data set). **b**. Sequence alignment of the CNBD of SKOR, KAT1, AKT1 and HCN1. The conserved arginine, which is important for cAMP binding, is highlighted in red. **c**. Structural comparison of CNBD in HCN1/cAMP complex (pink, PDB code 5u6p) and SKOR (blue). Potential clashes between cAMP and SKOR structure are indicated with black X.

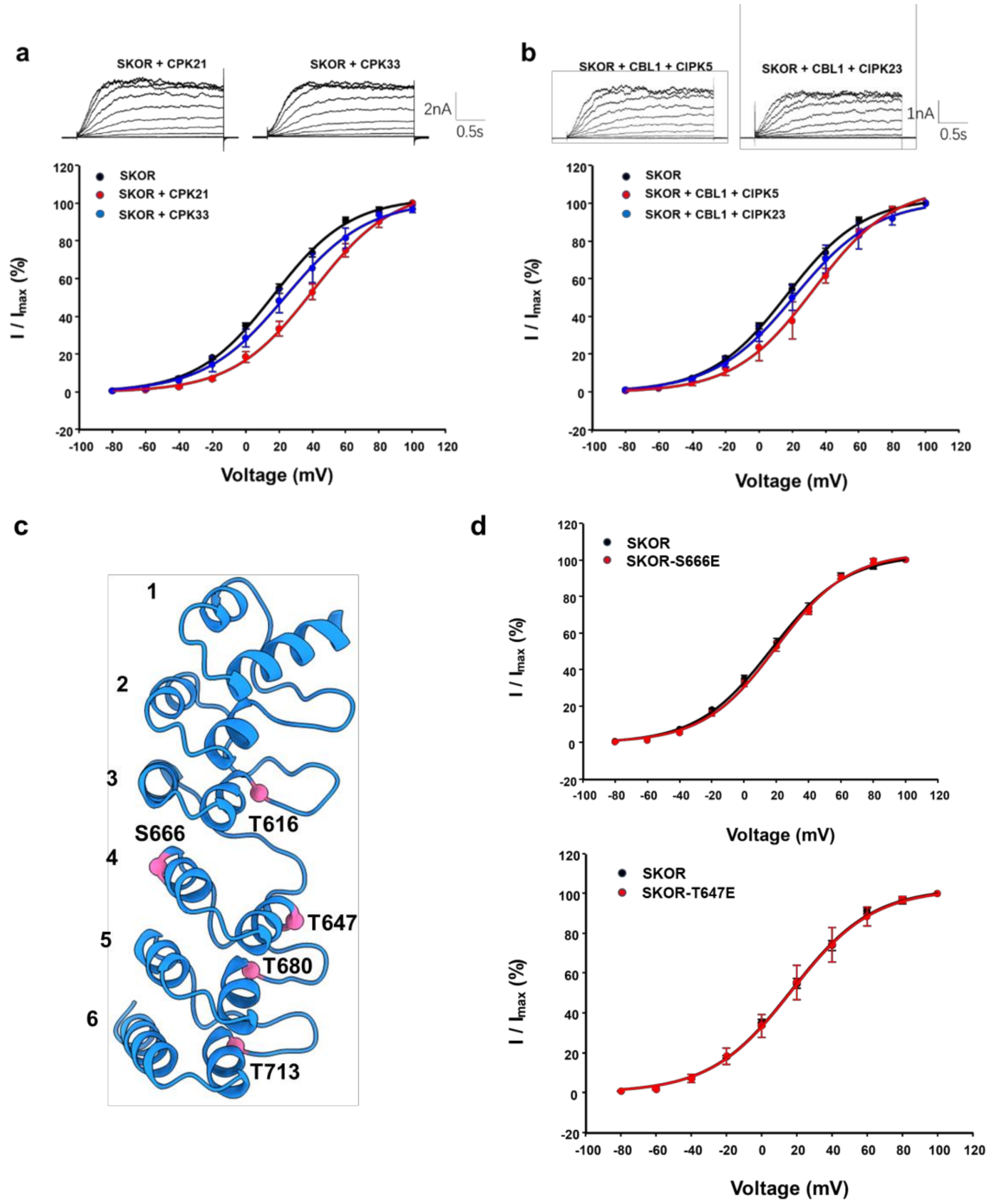

Supplementary Fig. S8. Function analysis of ankyrin-repeats domain of SKOR. **a.** Voltage dependence of the activation of SKOR channel (black), SKOR/CPK21 (red) and SKOR/CPK33 (blue). (means ±SE, n > 5 for each data set). **b.** Voltage dependence of the activation of SKOR channel (black), SKOR/CBL1/CIPK5 (red) and SKOR/CBL1/CIPK23 (blue). (means ±SE, n > 3 for each data set). **c.** Structure of ankyrin-repeats domain in SKOR. **d.** Voltage dependence of the activation of SKOR channel (black), the S666E mutant (red) (top) and the T647E mutant (red) (bottom). (means ±SE, n > 3 for each data set).

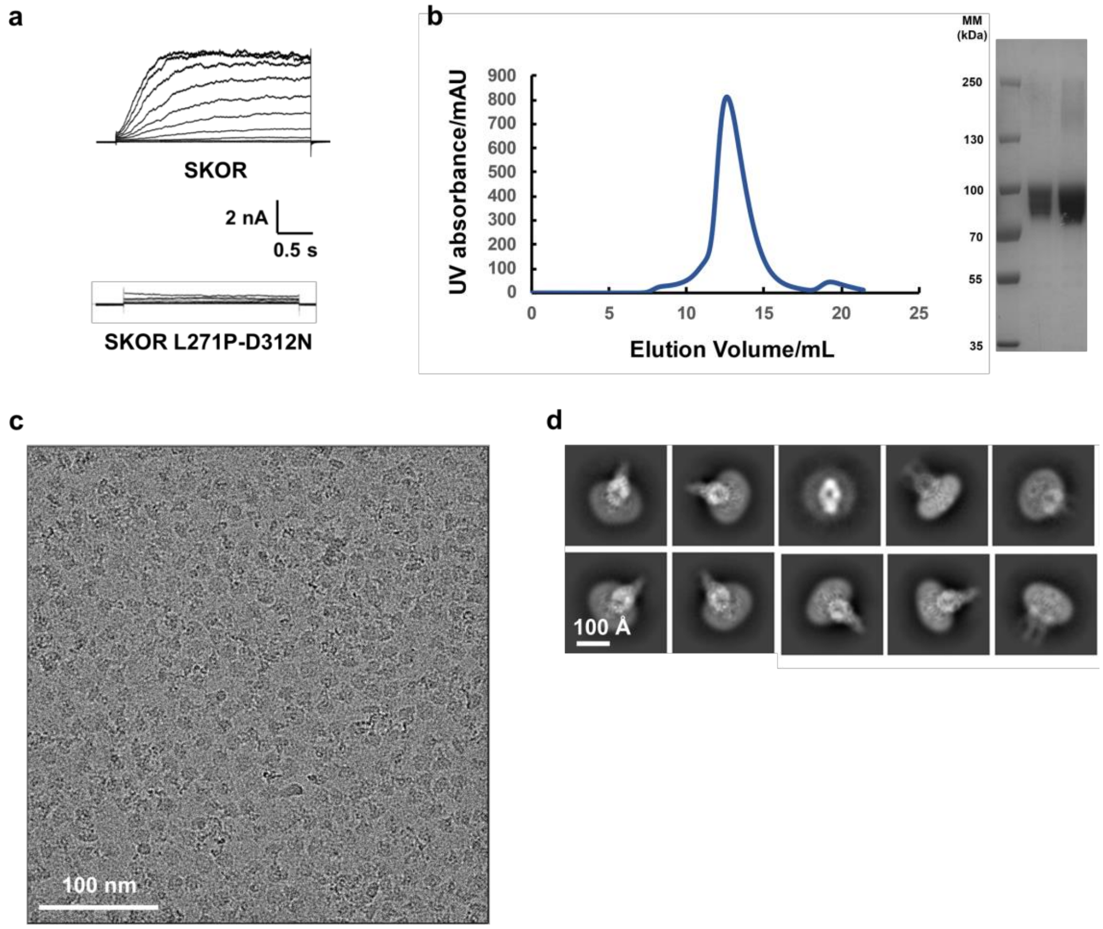

Supplementary Fig. S9. Purification and cryo-EM sample preparation of the SKOR L271P-D312N mutant. a. Whole cell currents of SKOR and the L271P-D312N mutant channels. **b**. Size-exclusion chromatography (left) and SDS-PAGE analysis (right) for purification. **c** and **d**. A representative cryo-EM micrograph (**c**) and representative 2D class averages (**d**) of the mutant.

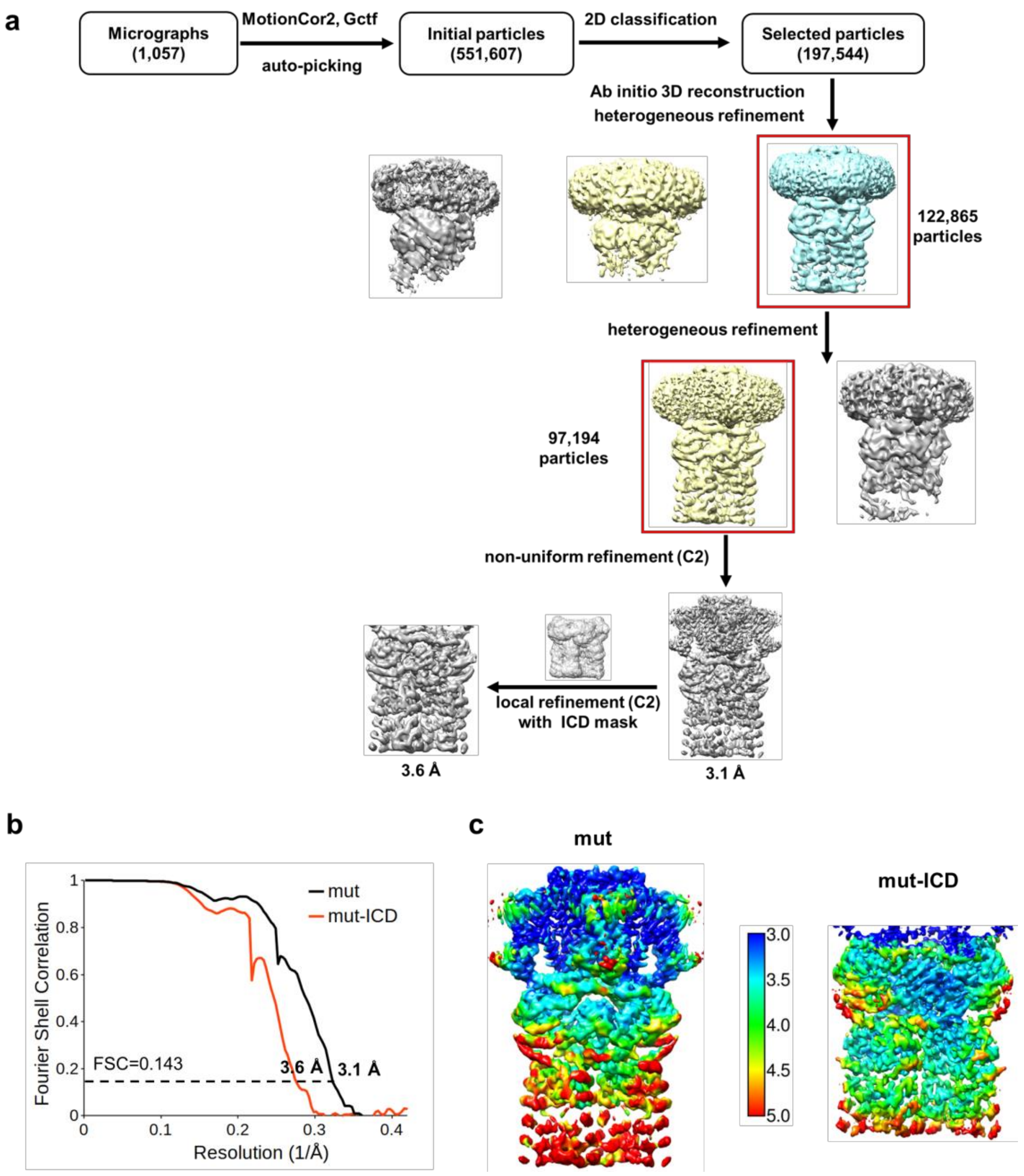

Supplementary Fig. S10. Cryo-EM structure determination of the SKOR L271P-D312N mutant. a. Cryo-EM data processing flow chart. **b**. Gold-standard Fourier shell correlation (FSC) curves for the 3D EM reconstructions of the mutant. **c**. EM density colored according to local resolution estimate.

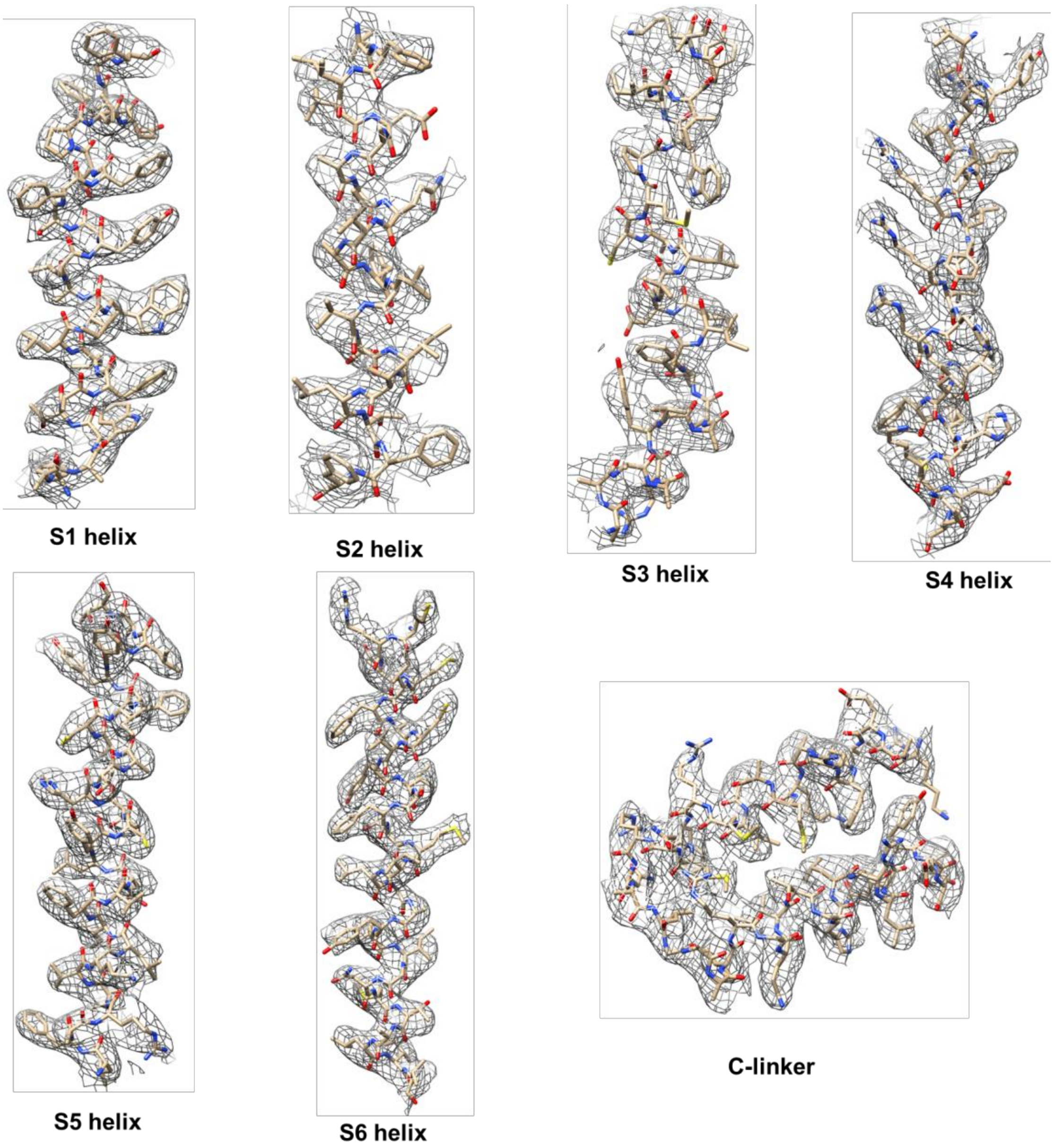

Supplementary Fig. S11. Agreement between the cryo-EM map and the model of the SKOR L271P-D312N mutant. Representative cryo-EM densities and fitted atomic models are shown.

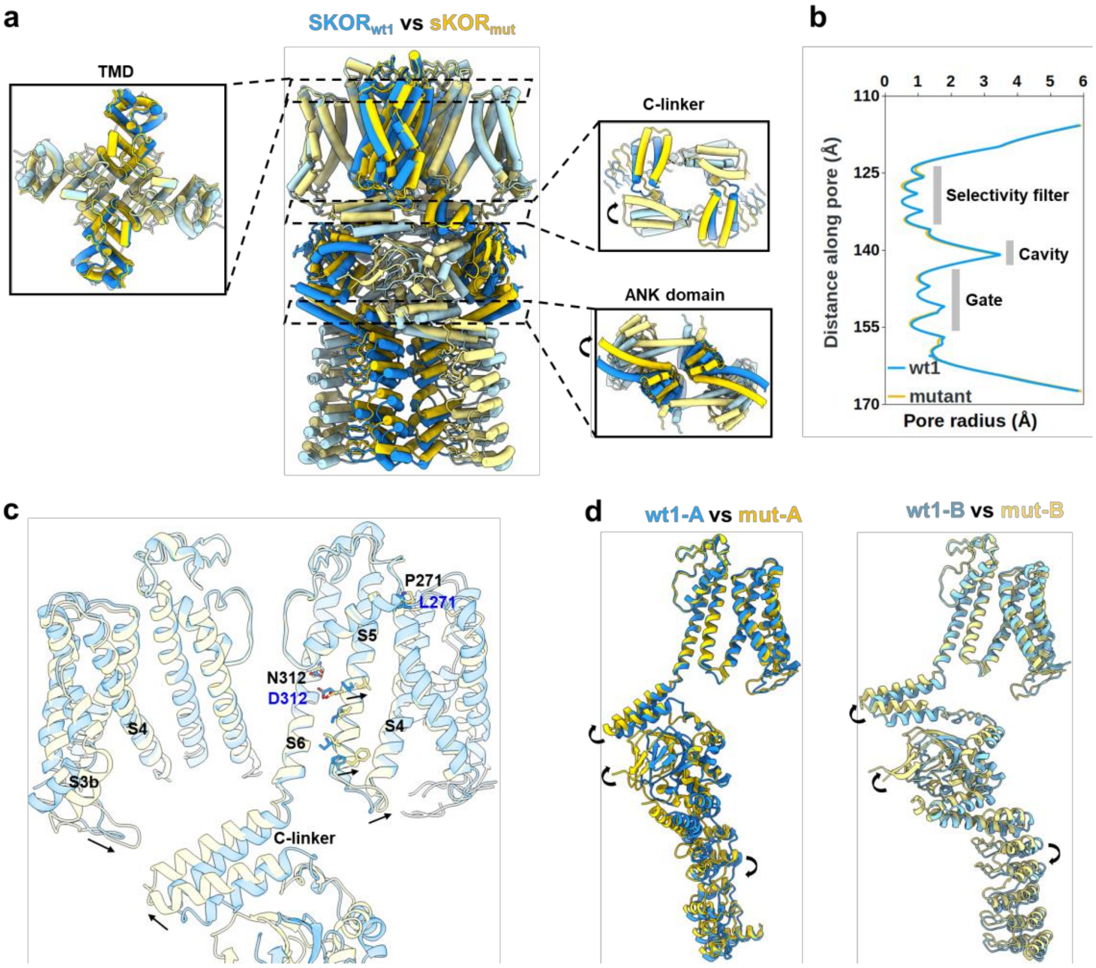

Supplementary Fig. S12. Structural comparison of SKOR$_{wt1}$ and the L271P-D312N mutant. a. Structural comparison of SKOR$_{wt1}$ (blue) and the mutant (yellow) reveals a rotation in the intracellular domains. **b**. Channel pore radius along the ion conduction pathway of SKOR (wt1: blue, mutant: orange) calculated using the HOLE program. **c**. Superimposition of the pore domain in SKOR$_{wt1}$ (blue) and the mutant (yellow) shows the structural differences in VSD and the ICD. The amino acids in position 271 and 312 are labeled. **d**. Overlay of a single subunit in SKOR$_{wt1}$ and the mutant.

Supplementary Table S1. Statistics of cryo-EM data collection, 3D reconstruction, model refinement and validation.

	$SKOR_{wt1}$ (EMDB-36195) (PDB 8JET)	$SKOR_{wt2}$ (EMDB-36196) (PDB 8JEU)	$SKOR_{mut}$ (EMDB-36185) (PDB 8JEC)	$SKOR_{wt1\text{-}ICD}$ (EMDB-36197)	$SKOR_{wt2\text{-}ICD}$ (EMDB-36198)	$SKOR_{mut\text{-}ICD}$ (EMDB-36199)
**Data collection and processing**						
Magnification	29,000	29,000	29,000	29,000	29,000	29,000
Voltage (kV)	300	300	300	300	300	300
Electron exposure ($e^-/Å^2$)	56	56	56	56	56	56
Defocus range (μm)	-1.0~-2.0	-1.0~-2.0	-1.0~-2.0	-1.0~-2.0	-1.0~-2.0	-1.0~-2.0
Pixel size (Å)	1.01	1.01	1.01	1.01	1.01	1.01
Symmetry	C2	C2	C2	C2	C2	C2
Initial particle images (no.)	765,927	765,927	551,607	765,927	765,927	551,607
Final particle images (no.)	129,992	62,707	97,194	129,992	62,707	97,194
Map resolution (Å)	3.1	3.5	3.1	3.9	4.0	3.6
FSC threshold	0.143	0.143	0.143	0.143	0.143	0.143
**Refinement**						
Initial model used	$SKOR_{mut}$	$SKOR_{mut}$	7ACL			
Model resolution (Å)	3.6	3.9	3.5			
FSC threshold	0.5	0.5	0.5			
Map sharpening *B* factor ($Å^2$)	-101.3	-96.3	-96.1	-170.0	-165.8	-138.2
Model composition						
Non-hydrogen atoms	19,526	19,438	19,680			
Protein residues	2612	2640	2632			
Ligands						
B factors ($Å^2$)						
Protein	144.32	147.40	136.96			
Ligand						
R.m.s. deviations						
Bond lengths (Å)	0.002	0.002	0.002			
Bond angles (°)	0.430	0.518	0.402			
Validation						
MolProbity score	1.67	1.75	1.56			
Clashscore	3.61	6.95	4.85			
Poor rotamers (%)	1.54	0.17	1.03			
Ramachandran plot						
Favored (%)	94.55	94.70	95.75			
Allowed (%)	5.45	4.99	4.17			
Disallowed (%)	0.00	0.30	0.08			
